# DEVELOPING CONTENT FOR A VIRTUAL SOCIAL ENGAGEMENT INTERVENTION FOR PERSONS WITH MCI

**DOI:** 10.1093/geroni/igad104.0320

**Published:** 2023-12-21

**Authors:** Wendy Rogers, Shraddha Shende, Sarah Jones, George Mois, Elizabeth Lydon, Raksha Mudar

**Affiliations:** University of Illinois Urbana-Champaign, Champaign, Illinois, United States; Illinois State University, Normal, Illinois, United States; University of Illinois-Urbana Champaign, Champaign, Illinois, United States; University of Illinois Urbana Champaign, Champaign, Illinois, United States; University of Illinois Urbana-Champaign, Champaign, Illinois, United States; University of Illinois Urbana-Champaign (UIUC), Champaign, Illinois, United States

## Abstract

Older adults, especially those with mild cognitive impairment (MCI), are at increasing risk of social isolation and loneliness, leading to poorer health outcomes. Video technology has the potential to provide socially and cognitively engaging activities from their homes. We are exploring the benefits of a virtual social engagement intervention in older adults with and without MCI (clinicaltrials.gov NCT05380180). We are using a video-technology called OneClick.chat to present participants with a short presentation followed by conversational breakout groups. One challenge for the intervention development was the selection of content that would be engaging but not too memory demanding and of interest to a broad range of people. We worked with iN2L to design 60 unique events that met predefined criteria: could facilitate conversations, did not rely on episodic memory, covered a range of topics, and reflected a diversity of individuals, identities, cultures, and interests. Events were categorized into five predefined categories: Arts & Culture; Nature, Health, & Wellness; Life Experiences; Science & Technology; and Recreation & Sports. We carefully curated each event to ensure consistency of presentation length, picture type, readability, visibility, number of slides, and audio quality. Presentation format was carefully tested through multiple iterations to ensure the best participant experience. Conversational prompts were designed for each event to stimulate engaging conversations through open-ended questions. During the trial we are gathering data on participants’ preferences for different topics that can guide future iterations. Our systematic process of development can guide content design for research on social engagement interventions.

